# Time-course analysis system for leaf feeding marks reveals effects of Arabidopsis trichomes on insect herbivore feeding behavior

**DOI:** 10.1093/jxb/erae184

**Published:** 2024-04-22

**Authors:** Naoyuki Sotta, Toru Fujiwara

**Affiliations:** Department of Applied Biological Chemistry, Graduate School of Agricultural and Life Sciences, The University of Tokyo, Tokyo 113-8657, Japan; Department of Agricultural Biology, Graduate School of Agriculture, Osaka Metropolitan University, Osaka 599-8531, Japan; Department of Applied Biological Chemistry, Graduate School of Agricultural and Life Sciences, The University of Tokyo, Tokyo 113-8657, Japan; University of South Bohemia in České Budějovice, Czech Republic

**Keywords:** *Arabidopsis thaliana*, defense, image analysis, *Pieris rapae*, plant–herbivore interactions, *Spodoptera litura*

## Abstract

Bioassay with an insect herbivore is a common approach to studying plant defense. While measuring insect growth rate as a negative indicator of plant defense levels is simple and straightforward, analysing more detailed feeding behavior parameters of insects, such as feeding rates, leaf area consumed per feeding event, intervals between feeding events, and spatio-temporal patterns of feeding sites on leaves, is more informative. However, such observations are generally time consuming and labor-intensive. Here, we provide a semi-automated system for quantifying feeding behavior parameters of insects feeding on plant leaves. Automated photo scanners record the time-course development of feeding marks on leaves. An image analysis pipeline processes the scanned images and extracts leaf area. By analysing changes in leaf area over time, it detects insect feeding events and calculates the leaf area consumed during each feeding event, providing quantitative parameters of the feeding behavior of insects. In addition, it visualizes spatio-temporal changes in feeding sites, providing a measure of the complex behavior of insects on leaves. Using this analysis pipeline, we demonstrate that Arabidopsis trichomes reduce insect feeding rate, but not feeding duration or intervals between feeding events. Our image acquisition system requires only a photo scanner and a laptop computer and does not require any specialized equipment. The analysis software is provided as an ImageJ macro and R package and is available at no cost. Taken together, our work provides a scalable method for quantitative assessment of the feeding behavior of insects on leaves, facilitating understanding of plant defense mechanisms.

## Teaser

An automated system was developed for time-course analysis of insect feeding marks on plant leaves, providing an accessible platform for studying plant defense mechanisms with quantitative parameters of insect feeding behavior.

## Introduction

For studies on plant defense systems against insect herbivores, bioassay is a fundamental and direct method to evaluate plant defense levels. Bioassay with herbivorous insects can be broadly classified into two categories, choice and non-choice experiments. In choice experiments, insects are given food choices, such as plants with different genotypes or treatments. The frequency of their choices is measured as an indicator of their preference, and thus, inversely, the level of plant defense. In non-choice experiments, insects are fed plants with different genotypes or treatments, and their growth rate is measured as a negative indicator of plant defense levels. Growth rate is a critical factor in insect survival, as a prolonged larval stage generally increases the risk of predation or parasitism, which inversely benefits plants.

The growth rate of insects does not solely reflect the levels of plant defense. For instance, the feeding behavior of *Pieris rapae* (cabbage white butterfly) affects parasitism by *Cotesia glomerata* (a parasitoid wasp) (Nakayama *et al.*, 2018). When not feeding, young *P. rapae* larvae rest at a short distance away from their feeding sites. This behavior has been demonstrated to be adaptive in terms of reducing the risk of parasitism by *C. glomerata*, which seeks hosts near the feeding site on leaves by detecting chemicals around the wounds caused by feeding leaves. Thus, to understand plant defense adaptation in natural environments in the presence of insect herbivores, it is crucial to consider detailed parameters of insect feeding behavior, not only simple growth rate.

However, the analysis of detailed feeding behavior of insects, such as feeding/resting time, amount of leaf consumed per feeding event, and spatial patterns of feeding marks, is often labor-intensive. A simple method to obtain these parameters is manual time-course observations (Nakayama *et al.*, 2018), but this is time-consuming and sometimes practically impossible depending on the scale of the experiment. In this regard, digital image analysis is a possible solution. Although various protocols and programs for leaf detection and leaf area quantification from digital images have been reported (Baur *et al.*, 1990; Hagerup *et al.*, 1990; Varma and Osuri, 2013; Easlon and Bloom, 2014; Machado *et al.*, 2016; Tech *et al.*, 2018), only a few enable temporal analysis of insects feeding behavior. Ji *et al.* (2017) proposed digital video-based time-lapse imaging of leaf area consumed by insects, and their MatLab-based algorithm characterized detailed feeding parameters of *Spodoptera litura* larva on sweet pepper leaf, such as bout time, interbout intervals, and leaf mass of each bout. Although this approach provides quantitative parameters of insect feeding behavior, its throughput has been limited, and it has not been applied for comparison among plant genotypes or treatments. Recently, a webcam-based high-throughput bioassay method has been proposed to analyse the feeding behavior of 150 larvae simultaneously (Sanané *et al.*, 2021). A webcam records images of leaf discs in small cages containing insects, and the computer program calculates the leaf disc area consumed by the insects over time. By analysing the time-course data with an algorithm, it classifies the feeding behavior of insects into six categories. While this method is effective for classifying the behavior of a large number of insects, it does not provide quantitative parameters such as the leaf area consumed by each feeding event and the interval between feeding events. In addition, the method is designed for leaf discs and application to intact leaves is not mentioned.

Here, we established a quantitative time-course measurement system for caterpillar feeding behavior with a throughput suitable for characterizing genetic differences in plants defense against insects. Our automated scanning system and image analysis algorithm enabled quantification of leaf area consumed by insects over time, providing quantitative parameters of feeding behavior, including leaf area consumed in a feeding event, duration of feeding, and intervals between each feeding. We also developed a program for spatio-temporal visualization of feeding patterns, which enables the study of differences in spatial patterns of feeding marks.

As a case study to quantitatively evaluate the effect of plant defense systems on insect behavior, we investigated effect of Arabidopsis trichomes on feeding parameters of chewing herbivores. In plant defense, trichomes are generally known to function as physical barriers against insect herbivores (Bar and Shtein, 2019). Different types of trichomes have different mechanisms to protect plants from insects: some trichomes secrete chemicals (glandular trichomes) to affect insect behavior, and others (non-glandular trichomes) with needle- or hook-shape reduce insect movement (Wagner *et al.*, 2004). A study on the effect of *Solanum carolinense* (horsenettle) trichomes on *Manduca sexta* (tobacco hornworm) illustrated that its non-glandular trichomes inhibit caterpillar growth not only via a pre-ingestive effect as a physical barrier to deter feeding, but also via a post-ingestive effect to damage the caterpillars’ digestive system (Kariyat *et al.*, 2017). The defensive function of non-glandular trichomes is also demonstrated in the model plant Arabidopsis. *Pieris rapae* fed on the trichome-less *gl1* mutant gained more weight than those fed on the wild type (Reymond *et al.*, 2004). Although it is considered that Arabidopsis trichomes hamper insects feeding as a physical barrier, to our knowledge, the detailed mechanism of the growth inhibition has been unclear, especially about which parameter of insect feeding behavior is affected by Arabidopsis trichomes.

Here, our study illustrates the effect of Arabidopsis trichomes on insect feeding behavior and demonstrates the effectiveness of our analytical framework. The method is potentially applicable to various studies on plant defense system and insect behavior. The image analysis pipeline is implemented on open-source platforms, Fiji (ImageJ) (Schindelin *et al.*, 2012) and R (R Core Team, 2023), and is publicly available at GitHub (https://github.com/nsotta/feeding-mark-analysis).

## Materials and methods

### Plants and insect materials

To obtain fully expanded flat rosette leaves by avoiding contact with neighboring plants, Arabidopsis Col-0 wild type and *gl1-1* (CS28175) were grown with a single seedling per pot. Seeds were sown on rockwool blocks placed on vermiculite. Pots were placed in an incubator at 22 °C, short day (8 h light/16 h dark) light conditions (80–150 μmol m^−2^ s^−1^), and 70% humidity. Pots were watered when the surface of the vermiculite dried. For nutrition, MGRL medium (Fujiwara *et al.*, 1992) was supplied once a week.

To obtain *Pieris rapae* larvae, adult butterflies were collected from fields in Tokyo or Yamanashi, Japan. Adult butterflies were reared in a net cage with pots of Chinese cabbage, and eggs were laid on the Chinese cabbage leaves. Larvae were reared on the Chinese cabbage leaves. Eggs of *Spodoptera litura* were purchased from Sumika Technoservice Corp. (Takarazuka, Japan), and larvae were reared on artificial diet Insecta F-II (Nosan, Japan). The larvae were starved for 3 h before the assay.

### Experiment set up and image acquisition

The experiment was conducted in an air-conditioned room maintained at 23 °C. Transparent, colorless styrol boxes with lids measuring 65 × 34 × 18 mm (Sanplatec, Japan) were used as chambers for the feeding assay. The size is suitable for placing a single mature Arabidopsis rosette leaf. The styrol boxes were aligned on a scanner (GT-X970 or GT-X980, Epson, Japan). With an A4-size scanner, 24 (6 × 4) leaf samples can be scanned at once. Mature flat leaves of Arabidopsis were cut at the base of the petiole with scissors and fixed to the bottom of the styrol box with the adaxial side up using paper tape. The cut end of the petiole was covered with moist paper tissue to prevent dehydration during the assay. To start the assay, a young larva of *P. rapae* or *S. litura* was placed on the leaf in the styrol box using a soft fine paintbrush, and the lid was placed on the styrol box to prevent the larva from escaping. For reflective light scanning mode, the whole scanning area was covered with blackout cloth to make the background of the scanned images black. For transmissive scanning mode, the light source panel of the scanner was placed on the scanning area. Images were scanned at 266–600 dpi every 1 min, and the images were saved as JPEG files with the serial frame number in their filename suffix. The scanner was controlled from a laptop computer by the scanner driver software, and the operation was automated by the UWSC keyboard/mouse macro tool (https://www.vector.co.jp/soft/winnt/util/se115105.html).

### Leaf area quantification by image analysis

Image analysis was performed using Fiji with a custom macro. The JPEG files of each time point were pre-processed one by one before quantification. In the first step, leaves, larvae, and feces were detected using the ‘Color threshold’ macro, and the resulting mask was saved as ‘mask1’. To exclude the pixels derived from larvae or feces from the leaf area quantification, cumulative time-difference masking was applied to the mask1 images. This algorithm takes the difference between two consecutive frames (frame *t* and *t*+1, starting from *t*=1) and updates frame *t*+1 by converting all pixels that were background in frame *t* into background pixels. Assuming that pixel values change only when a leaf is consumed or an insect moves, and that once a leaf part is consumed, that part will never return to its original state, this algorithm can mask false-positive leaf pixel detection caused by insect bodies similar in color to the leaf. The resulting images were saved as ‘mask2’. The region of interest (ROI) for each cage was defined by manually selecting a rectangular region containing an entire leaf blade and saved as a zip-compressed ImageJ ROI file. Leaf area for each time frame was calculated for each ROI against mask2, and the measured area values were saved as CSV files.

### Calculation of parameters of insect feeding behavior

To calculate the parameters of the feeding behavior from the leaf area time series data, the CSV files are loaded and processed by our R package feedingMarkAnalyzeR. It loads all CSV files that match the file name pattern specified by the user and sorts them by frame number and the ROIs, resulting in leaf area as a function of time [*S*_leaf_(*t*)]. For each ROI, the time derivative of the leaf area [Δ*S*_leaf_(*t*)] was calculated as follows:


$$\begin{array}{l} \Delta S_{leaf}\left(t\right)=S_{leaf}\left(t+1\right)-S_{leaf}\left(t\right) \end{array}$$

where *S*_leaf_(*t*) represents the leaf area at frame=*t*. The feeding behavior was visualized by plotting *y*=−Δ*S*_leaf_(*t*), where each feeding event is represented by peaks. The regions where consecutive data points exceed a threshold value (default 0.2 mm^2^ min^−1^) are detected as peaks. For each peak (=feeding event) the following parameters were calculated: leaf area consumed (the integrated area of the peak, mm^2^), feeding time (the width of the peak, min), average feeding rate during each feeding event (mm^2^ min^−1^). The intervals between each feeding event (min) were calculated by taking the distance from the end of one peak to the beginning of the next peak. Intervals longer than 100 min were excluded from the calculation of feeding intervals because they reflect molting in most cases. ROIs with apparent false-positive detection of feeding marks due to accidental leaf movement or feces on the leaf are omitted from the analysis.

### Color composition analysis

The images of the leaf with larvae scanned in reflective mode were opened in GIMP (https://www.gimp.org), and the background, larvae, and leaf regions were manually separated and saved as separate TIF files. The saved images were opened in Fiji, and color composition analysis was performed using the Color Inspector 3D plugin with RGB color space and display mode: All Colors.

### Visualization of feeding marks

PNG images of the leaf masks were loaded into R and processed using the imager package (Barthelme, 2023). To visualize the feeding mark formed during each feeding event, image masks of feeding marks were created for each feeding event detected in the upstream analysis by taking the differences between the leaf detection masks of one frame before the start and the last frame of each feeding event. The first frame of the leaf area mask was used as the reference, and leaves are represented as black. For each feeding event, the color of pixels corresponding to the feeding mark formed by the current feeding event was replaced with magenta, and those corresponding to old feeding events were replaced with magenta.

To extract the coordinates of each feeding mark, the center of gravity of each feeding mark was calculated. The Euclidean distances between feeding marks of two consecutive feeding events were calculated. A data table containing the order, coordinates, and start/end time of each feeding mark and the distance from the previous feeding mark was exported as a CSV file. The temporal changes in feeding mark coordinates and distances from the previous feeding mark were visualized using the ggplot2 package (Wickham, 2016).

## Results

### Establishment of an automated system for recording insect feeding behavior on leaves

**Fig. 1.**
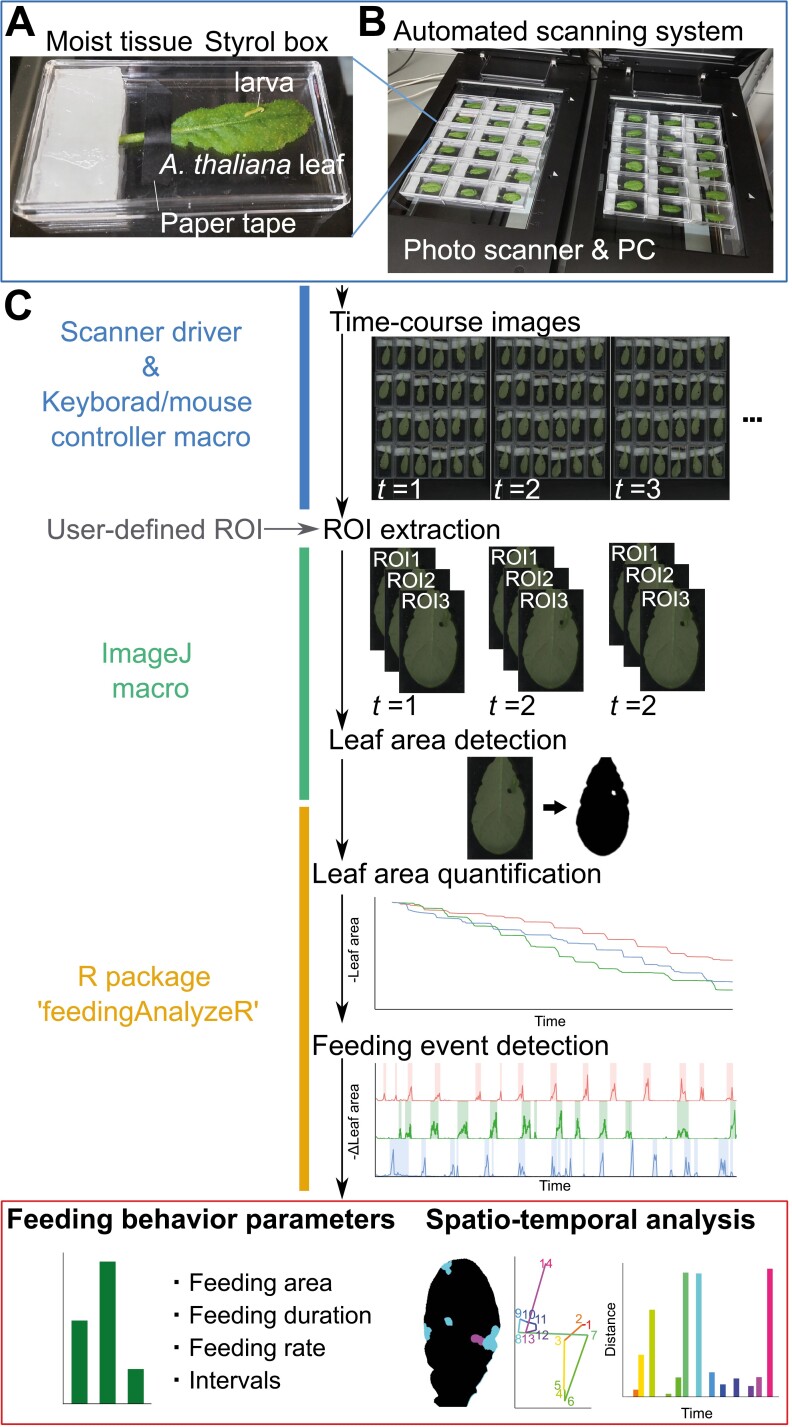
Experimental set up and workflow for semi-automated acquisition of insect feeding behavior parameters on leaves. (A) Plastic cage set up for assay. An Arabidopsis leaf is attached to the bottom of a plastic cage (W 65 mm, D 34 mm, H 18 mm) with paper tape. (B) Scanner set up for data collection. The cages were placed on the photo scanners and scanned from below. (C) Workflow of image acquisition and data processing.

To facilitate a detailed assessment of the mechanisms of plant tolerance to insects, we developed a system to quantify the parameters of insect feeding behavior on plant leaves. The system consists of an automated scanning system to obtain time-lapse images of formation of feeding marks on leaves, and an image analysis pipeline that processes the scanned image to detect changes is leaf area over time (Fig. 1). Leaves are subjected to feeding by an insect herbivore in a plastic cage and scanned over time by a photo scanner (Fig. 1A, B). The image analysis pipeline first detects the leaf area for each time frame. From the changes in the leaf area over time, the program detects each feeding event and outputs feeding parameters: leaf area consumed per feeding, duration of feeding, rate of leaf area consumption during one feeding event, and intervals between feeding events (Fig. 1C). It also provides spatio-temporal information on the formation of feeding marks, enabling visualization of insect feeding behavior on leaves.

### Time-course analysis of feeding mark images provides herbivore feeding behavior parameters

**Fig. 2.**
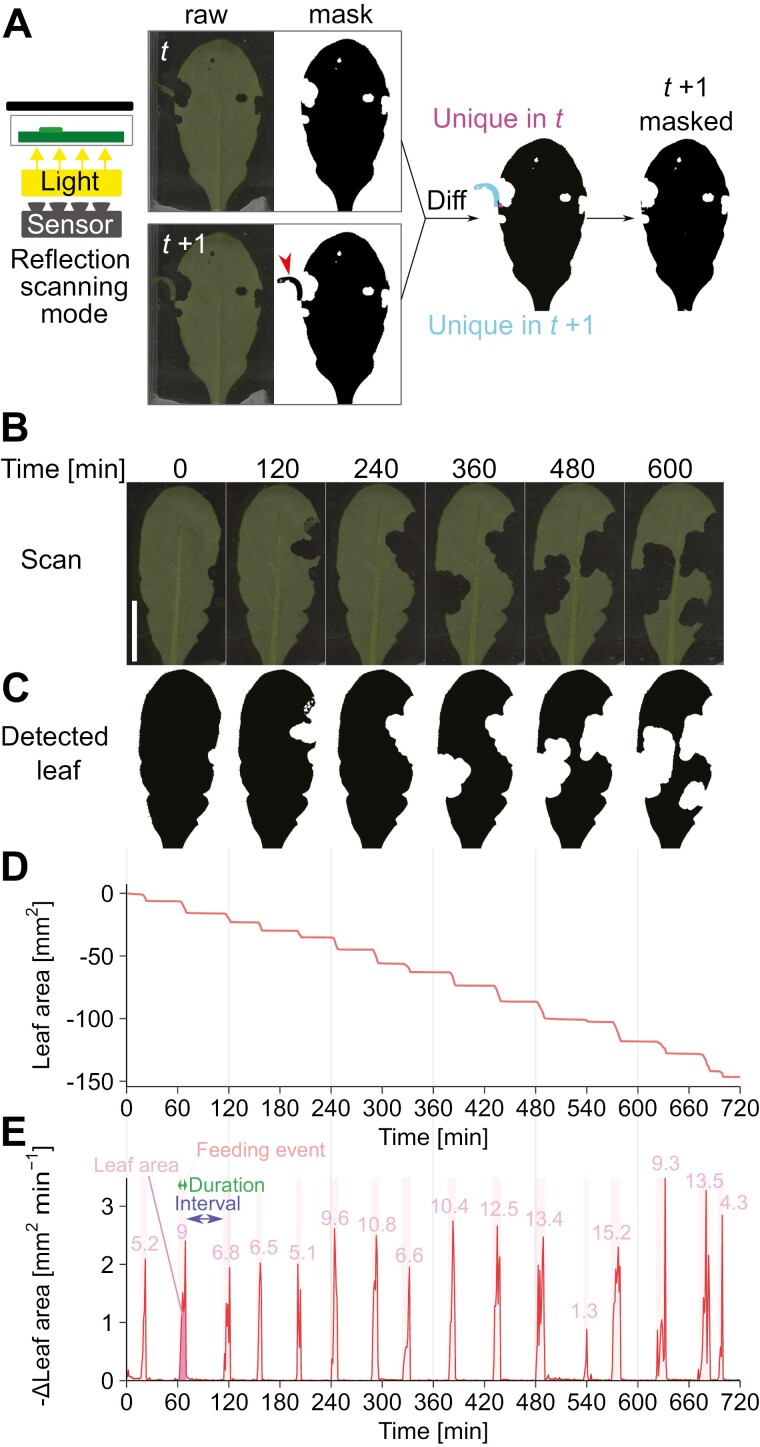
Image analysis for detection and quantification of insect feeding events on leaves. (A) Scheme of image analysis for leaf detection. The raw images were binarized by color thresholding to detect leaf area. False positive detection caused by insect body was filtered by masking the images with that of the previous time frame. (B) Examples of leaf images acquired by the scanner over time. (C) Leaf area detected by image analysis. (D) Changes in leaf area over time detected by image analysis. Leaf area at *t*=0 is set to zero. (E) Detection of feeding events from the time derivative of the leaf area time function [Δ*S*_leaf_(*t*)]. Feeding events are observed as peaks, and the leaf area consumed is expressed as the integral area of each peak. The numbers to the right of the peaks represent the peak area in mm^2^.

Here we illustrate how this system quantifies feeding behavior with an example of *P. rapae* feeding on Arabidopsis leaves. A *P. rapae* larva was fed on a single Arabidopsis leaf placed on the bottom of a transparent plastic cage and scanned by a photo scanner from below in reflective scanning mode (Fig. 2A). The scanner was automatically operated by mouse/keyboard macro software, which allowed time-lapse scanning with 1 min interval for more than 12 h without attendance.

The resulting image series were then analysed by the image analysis pipeline. To calculate the leaf consumption by the insect over time, the program first detected the leaf area for all time frames. By applying a color threshold to the acquired image, it successfully distinguished leaves from the background (Fig. 2A). However, in some cases, color thresholding alone was unable to distinguish leaves from the larva body due to the similarity of their colors (Fig. 2A, red arrowhead). To exclude the larva from leaf area detection, we implemented cumulative time-difference masking (Fig 2A). Under the assumption that once a pixel becomes a background pixel, it never becomes a leaf pixel again, it successfully excluded non-leaf pixels from leaf detection (Fig. 2A, ‘masked’). This processing algorithm was implemented as an ImageJ macro and can be executed by simply selecting the input and output directories, and a user-defined region of interest (ROI) file from the graphical user interface.

By quantifying the area of the detected leaves for each time frame, the temporal changes in leaf area [Δ*S*_leaf_(*t*)] were obtained for each assay (Fig. 2B–D). The time profile of leaf area exhibited a stepwise decrease, reflecting the intermittent feeding behavior of the larvae. To detect feeding events from the obtained leaf area profile, we calculated the differences of leaf areas between two consecutive time frames [Δ*S*_leaf_(*t*)] (Fig. 2E). In the *y*=−Δ*S*_leaf_(*t*) plot, the non-feeding state has values around zero, while the feeding state is expressed as a peak. The area of each peak represents the leaf area consumed by the larva during a single feeding event. To detect and quantify each feeding event, we implemented a peak detection algorithm that detects regions where consecutive data points exceed a threshold. By selecting an appropriate threshold, it successfully detected each feeding event (Fig. 2E, highlighted in red). Peak detection allowed the calculation of quantitative parameters of feeding behavior, such as duration, leaf area consumed, and feeding rate for each feeding event, as well as intervals between feeding events. We implemented these data processing algorithms as an R package so that users can obtain quantified feeding parameters by running a few simple functions. Taken together, the scanning system and image processing pipeline we developed enable quantitative evaluation of insect feeding behavior on leaves.

### Arabidopsis trichomes affect *P. rapae* leaf consumption per feeding but not feeding interval

**Fig. 3.**
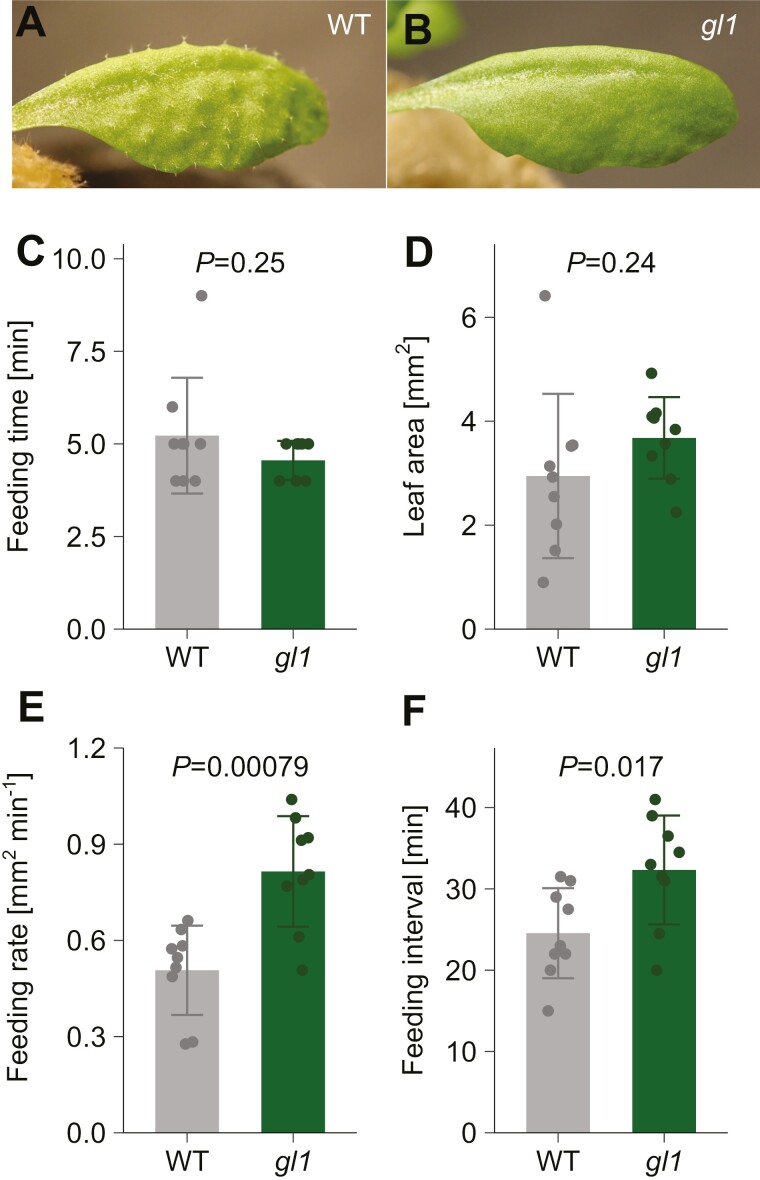
Quantified parameters of *P. rapae* feeding behavior on Arabidopsis. (A, B) Representative images of wild-type (A) and *gl1* (B) mature rosette leaf. (C–F) Feeding behavior parameters of *P. rapae* on Arabidopsis leaves quantified in the reflective scanning mode. For each assay, the median of all feeding events during 999 min was used as the representative value of the assay. The mean ±SD of nine assays is plotted. *P*-values shown are for differences between the wild type and *gl1* tested by Welch’s *t*-test. (C) Duration of each feeding event. (D) Leaf area consumed during each feeding event. (E) Leaf consumption rate during each feeding event. (F) Intervals between feeding events.

As a benchmark of the method, we sought to characterize the defensive effect of Arabidopsis trichomes against *P. rapae* larvae. To this end, we quantified the feeding parameters of *P. rapae* second instar larvae on trichome-less Arabidopsis mutant *gl1* (Fig. 3A, B) and compared them with those on wild-type plants. Using automated image acquisition and the image analysis pipeline, we obtained temporal profiles of leaf consumption with nine replicates for each genotype (Supplementary Fig. S1). Our algorithm successfully detected feeding events in all the assays, demonstrating its robustness.

Our analysis pipeline extracted four parameters of feeding behavior for each feeding event: leaf area consumed, duration, average feeding rate, and intervals from the last feeding event (Fig. 3C–F). While duration of each feeding event was comparable between the two genotypes (*P*=0.25, Welch’s *t*-test), the leaf area consumed per feeding event tended to be higher in the *gl1* mutant compared with the wild type, although it was not statistically significant at *P*<0.05 (*P*=0.24, Welch’s *t*-test). The difference was more pronounced in feeding rate (leaf area consumed per time during a feeding event), with a significantly higher feeding rate in *gl1* (*P*=0.00079, Welch’s *t*-test). In addition, the intervals between feeding events were significantly longer in *gl1* (*P*=0.017, Welch’s *t*-test). These results illustrate which parameters of insect feeding behavior are affected by Arabidopsis trichomes and provide insights into detailed mechanisms of the plant defense system.

### The analysis system is applicable to different insect species: *Spodoptera litura*

**Fig. 4.**
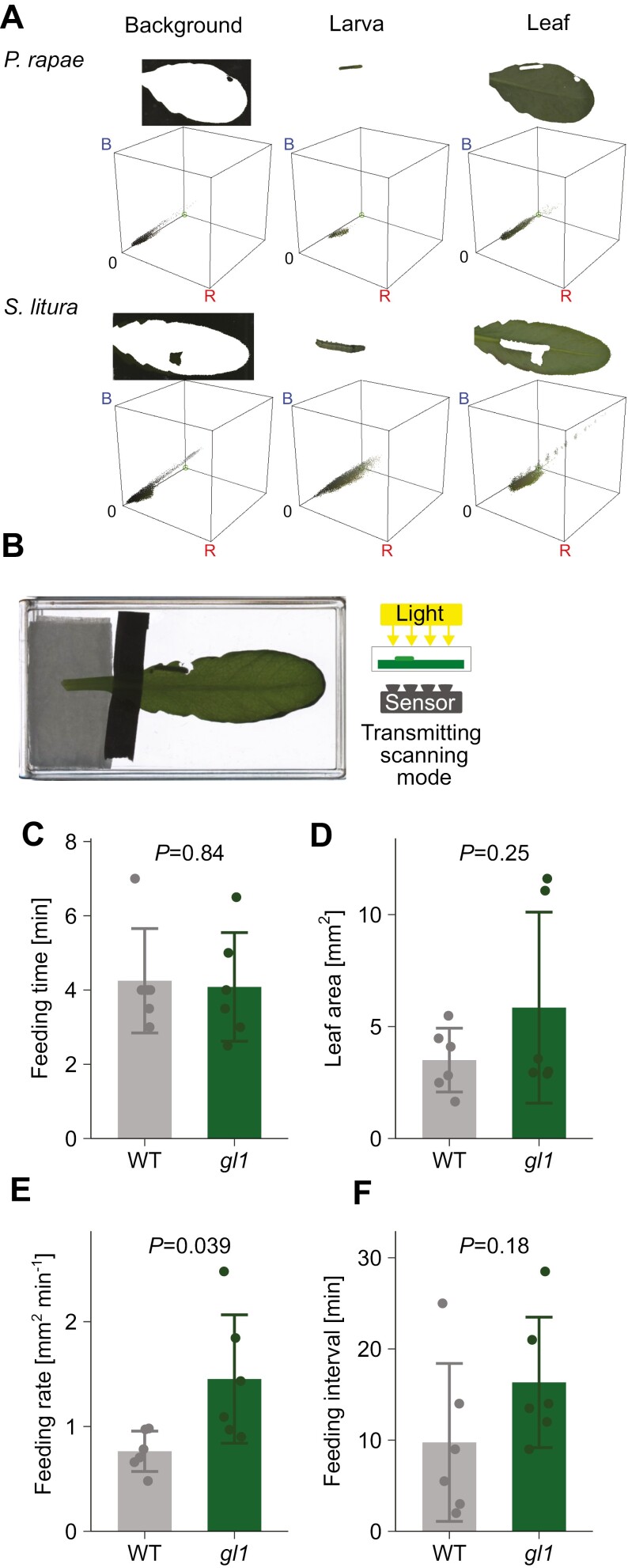
Quantified parameters of the feeding behavior of *S. litura* on Arabidopsis. (A) Body color composition of *P. rapae* and *S. litura*. In the images scanned in reflective mode, background, larva, and leaf regions were separated and subjected to color composition analysis. Colors present in each region are visualized in R (red), G (green), B (blue) color space. (B) An example of an image scanned in transmissive mode. (C, D) Feeding behavior parameters of *S. liture* on Arabidopsis leaves quantified in the transmissive scanning mode. For each assay, the median of all feeding events during 999 min was used as the representative value of the assay. The mean ±SD of six assays is plotted. *P*-values shown are for differences between the wild type and *gl1* tested by Welch’s *t*-test. (C) Duration of each feeding event. (D) Leaf area consumed during each feeding event. (E) Leaf consumption rate during each feeding event. (F) Intervals between feeding events.

To examine the versatility of our analysis pipeline, we analysed the feeding behavior of larvae of another species, *Spodoptera litura*, a generalist herbivore commonly used in experiments. Because their body color pattern is more complex with darker color components compared with *P. rapae* (Fig. 4A), it was expected that it would be technically difficult to separate their bodies from background or leaves using color thresholding. In fact, we were not able to specifically detect leaf area and feeding marks with the method used for *P. rapae*, because black patterns in *S. litura* were indistinguishable from black backgrounds, resulting in false-positive detection of feeding marks. To overcome this problem, we modified the scanning method. While *P. rapae* was scanned in reflective mode (light comes from the bottom, the same side as the scanning sensor), we adopted the transmissive light mode for *S. litura*. Illumination from the back produced an image with high contrast between the white background and the objects (Fig. 4B), enabling the separation of the leaf and insects from the background by color thresholding.

With the improved light settings, our scanning/image analysis system successfully obtained the time-course of feeding mark formation on Arabidopsis leaves by *S. litura*. As in the *P. rapae* experiment, we compared the effects of the Arabidopsis genotypes, WT and *gl1*, on the feeding behavior parameters of *S. litura* (Fig. 4C–F). The differences in feeding behavior parameters between the wild type and *gl1* was similar to the case of *P. rapae*. While no significant difference was observed in the duration of each feeding event, the feeding rate per feeding was significantly higher in the *gl1* mutant (Fig. 4E, *P*=0.039, Welch’s *t-*test). On the other hand, in contrast to *P. rapae*, the intervals between feeding events were not significantly different between genotypes, although they tended to be longer in *gl1* (Fig. 4F, *P*=0.18, Welch’s t-test). These results indicate that Arabidopsis trichomes have the effect of reducing feeding rate of at least two different herbivore species and demonstrate that our system is applicable to different herbivore species.

### Spatio-temporal visualization of feeding patterns

**Fig. 5.**
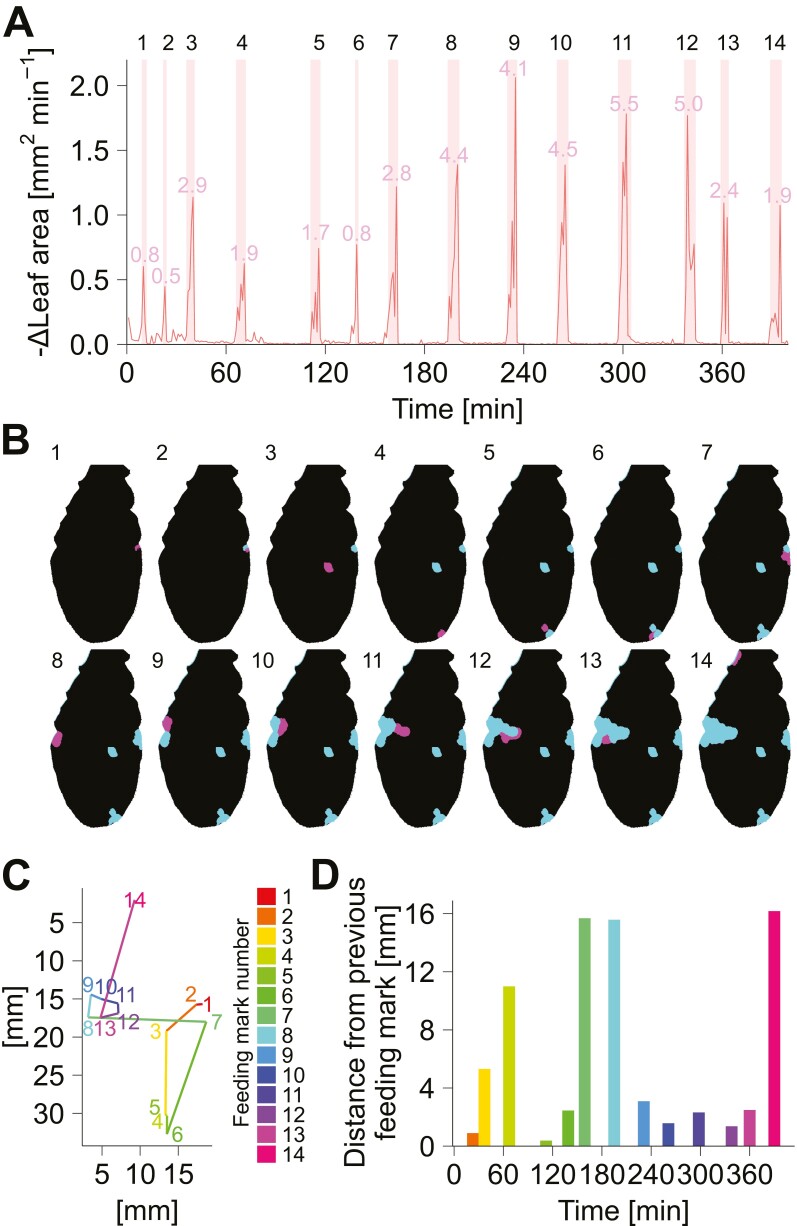
Visualization of the spatio-temporal pattern of *S. litura* feeding on an Arabidopsis leaf. (A) Visualization of leaf area reduction over time. The time derivative of leaf area is plotted. Feeding events are represented as peaks. Each feeding event detected by the thresholding algorithm is highlighted in red. (B) Visualization of the feeding marks formed by each feeding event. Numbers represent the order of feeding marks formed, corresponding to the peak numbers in (A). Black, remaining leaf area; magenta, leaf area consumed by the feeding event of interest; cyan, leaf area consumed by previous feeding events. (C) Summary plot of temporal changes in feeding position. The center of gravity of the leaf area consumed during each feeding event was calculated as the representative position of each feeding mark. The number and color represent the order in which the feeding marks were formed. (D) Distance from the previous feeding position. The colors correspond to the feeding mark number in (C).

To facilitate the analysis of spatio-temporal patterns of feeding behavior using our data acquisition platform, we have implemented functionality for positional analysis of feeding marks (Fig. 5). Using the masked images and the start/end time information of each feeding event, our visualization program highlights the leaf area consumed during each feeding event (Fig. 5A, B). It illustrates the spatio-temporal pattern of the insect feeding behavior, visualizing how the insect feeds on multiple distant feeding sites. The analysis pipeline also provides coordination of each feeding mark, enabling visual summary of the order of feeding sites (Fig. 5C). The example data highlighted that the *S. litura* larvae change their feeding sites regularly, sometimes returning to their previous feeding sites. The program also provides information on the distance between each feeding site, illustrating that feeding occurs sometimes near the previous site and sometimes at a distance from the previous site (Fig. 5D). Taken together, our system enabled a quantitative evaluation of the spatio-temporal feeding behavior of insects on leaves from automatically acquired image data.

## Discussion

### Availability, scalability, and applicability of the system

In this study, we established an affordable, semi-automated system for obtaining feeding behavior parameters of insect herbivores on plant leaves. Although previous studies have reported image analysis-based approaches for the analysis of insect feeding behavior (Ji *et al.*, 2017; Sanané *et al.*, 2021), to our knowledge, our system is the first to enable high-throughput acquisition of insect feeding parameters, including spatio-temporal data of insect feeding sites on intact leaves, which was not available in systems using leaf discs. The use of a photo scanner overcomes potential technical problems and low reproducibility in camera-based systems, including non-uniformity of the light source, differences in viewing angle, image distortion, and color calibration. Although our system requires computers for scanner control and image analysis, laptops for general use are sufficient. The image analysis involves only simple processing such as color thresholding and differential analysis, and thus does not require high computing power. This is an advantage over computationally intensive analysis methods such as those involving machine learning. The software developed in this study is built on ImageJ and R, both of which are among the most widely used open-source software for image analysis and statistical analysis. The programs are provided as an ImageJ macro and as an R package, feedingMarkAnalyzeR, and are available free of charge (https://github.com/nsotta/feeding-mark-analysis).

We propose two different scanning methods: the reflective mode used for *P. rapae* larvae, and the transmissive mode used for *S. litura.* The latter is more robust for leaf and insect detection because the illumination from above produces high contrast regardless of insect color, making background separation by color thresholding less challenging. Although we only tested *P. rapae* and *S. litura*, considering the high contrast between the object and the white background obtained in this mode, we assume that it enables the analysis of most insect herbivore species, except those with extremely white body colors. On the other hand, the former mode has the advantage of accessibility, since not all commercial photo scanners have the transmissive mode, and the function is generally limited to high-end models. If the target insects for the assay are larvae that do not contain dark colors, such as *P. rapae*, inexpensive scanners with reflective mode only will suffice.

Our measurement system is scalable in both hardware and software. The number of assays can be scaled by changing the number of cages and photo scanners. The number of assays per photo scanner depends on the size of the scanning area and the plastic cages. In our set up, an A4 size scanner allowed 24 assays with mature Arabidopsis leaves. The image analysis pipeline processes the image data per scanner, and the output of the pipeline can be merged for downstream analysis. This scalable feature enables experiments with large numbers of assay.

One limitation of scaling may be the scan interval. The shorter the interval, the higher the time resolution. However, the minimum interval is limited by the scanning speed of the scanner, because the image acquisition interval must be longer than the required scanning time. The time required to complete a single scan depends on the size of the scanning area and the scanning resolution, the type of image storage formats, and the specifications of the scanning device and the computer. In our set up, scanning an A4 area at 244 dpi in JPEG format allowed scanning with an interval of 1 min. The 1 min interval provided enough time-resolution for detecting each feeding event, as the typical duration of each event was 4~5 min in our observation (Figs 3C, 4C).

Another limitation could be the possible effect of detaching leaves from the plants. In the current form of the experimental set up, leaves must be cut at the petiole prior to assay. This makes it difficult to apply the method to studies that require intact plants, such as those on systemic responses. In addition, cutting the leaves may induce a wounding response in the plants, which could affect the behavior of the insects. However, these drawbacks are also present in existing methods using leaf discs (Sanané *et al.*, 2021). In this respect, the potential effect of wounding should be smaller in the present method due to the smaller cutting surface compared with leaf discs.

### Countermeasures for potential causes of false positive detection of feeding events

Long-time observation may result in shrinkage of leaves and reduction in leaf area. In addition, insect activity may result in changes in leaf position. These are potential cause of false-positive detection of leaf area consumed by the insects, because in our image algorithm feeding event is detected only using time-course changes in leaf area. In our system, these problems are addressed by both hardware and software countermeasure. Leaf shrinkage is prevented by moist tissue located on the cutting surface of petiole, and movement of leaves from their original position is prevented by paper tape that fixes the leaves to the bottom of plastic cages. In the image analysis, leaf shrinkage, if any, will be observed as a small reduction in leaf area over time. These slow changes in leaf area will not be detected as a feeding event, because the image analysis algorithm detects peaks in delta leaf area that are higher than a threshold. The threshold value can be changed when the user executes the function, which allows it to be adjusted accordingly for target experimental materials with different characteristics.

### Insights on defense mechanism of Arabidopsis trichomes against an insect herbivore

Although it has been suggested that Arabidopsis trichomes retard the growth of their predator (Reymond *et al.*, 2004), to our knowledge, the detailed mechanism by which trichomes suppress insect growth is not clear. One possibility is that Arabidopsis trichomes physically interfere with larval movement across the leaf, as has been reported in other plant species (Bar and Shtein, 2019). If this were the case, it would be expected that the larvae would require more time to forage, resulting in a longer interval between feeding events. However, in our observation for both *P. rapae* and *S. litura*, the interval was not longer (and even shorter in the case of *P. rapae*) in the wild type compared with the trichome-less *gl1* mutant. This suggests that the cause of slower growth of wild-type leaves compared with *gl1* leaves is not a prolonged foraging period. Rather, the most affected parameter in both herbivore species was feeding rate. The lower feeding rate in the wild type indicates that the insects can ingest less leaf in a certain period in the presence of trichomes than in their absence. On the other hand, the duration of each feeding event was not significantly affected by the presence of trichomes. These results may indicate that the duration of each feeding event is not determined by the amount of leaf ingested, but by how long they feed on a single site, and that trichomes retard insect growth by reducing the amount of leaf the insects ingest during the limited feeding time.

## Supplementary data

The following supplementary data are available at *JXB* online.

Fig. S1. Detection of feeding event of *P. rapae* feeding on Arabidopsis leaf.

erae184_suppl_Supplementary_Figure_S1

## Data Availability

The software and documentation for the analysis pipeline is available online (https://github.com/nsotta/feeding-mark-analysis).

## Abbreviation:

ROI: region of interest.
