# Supplementary material for: Time-course analysis system for leaf feeding marks reveals effects of Arabidopsis trichomes on insect herbivore feeding behavior
Source: J Exp Bot. 2024 Apr 22;75(17):5428–37. doi: 10.1093/jxb/erae184 (PMC11389832; doi:10.1093/jxb/erae184)
Supplement: erae184_suppl_Supplementary_Figure_S1 [file erae184_suppl_supplementary_figure_s1.pdf]

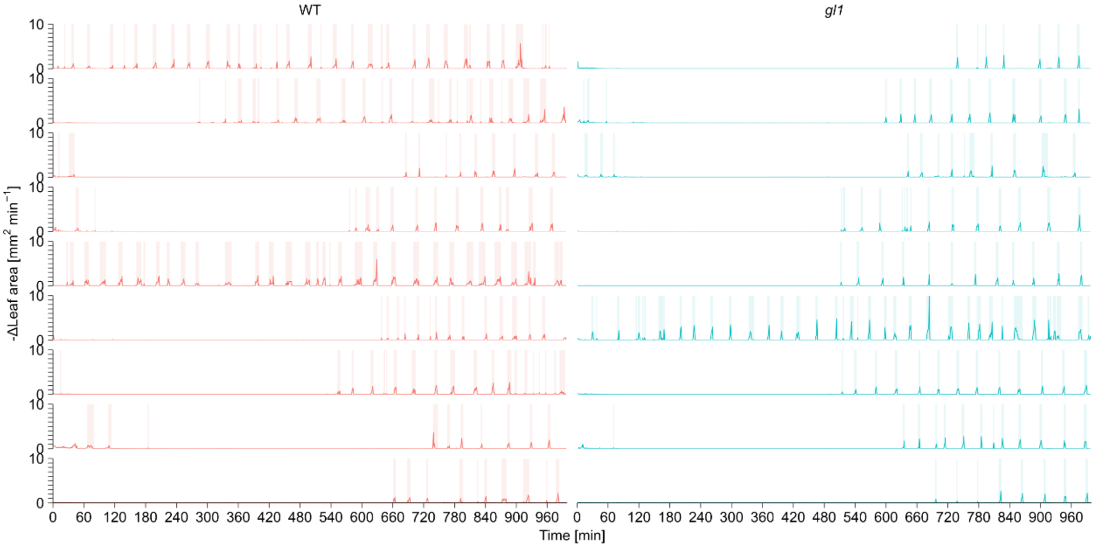

Figure S1. Detection of feeding event of *P. rapae* feeding on *A. thaliana* leaf.

Leaf area reduction over time from the assay with *A. thaliana* wild type and *gl1*. The time derivative of leaf area is plotted for nine replicates for each genotype. Feeding events are represented as peaks. Each feeding event detected by the thresholding algorithm is highlighted.
